# The Backwood’s-man in Philadelphia

**Published:** 1834

**Authors:** 


### X. The Backwood's-man in Philadelphia.

We are informed that Dr. M'Dowell's class on Anatomy and Surgery numbers 110 or 115—the largest we believe that was ever made up in that city, by a private lecturer. It is creditable to the professors of the two Philadelphia schools, that several of them, as we understand, have manifested towards him a decided liberality. Two or three years ago, a number of respectable citizens of this place, sought in vain, to procure for Dr. M'Dowell, the place of an *assistant* in the Medical College of Ohio!

